# The Effect of Adding CO_2_ to Hypoxic Inspired Gas on Cerebral Blood Flow Velocity and Breathing during Incremental Exercise

**DOI:** 10.1371/journal.pone.0081130

**Published:** 2013-11-21

**Authors:** Jui-Lin Fan, Bengt Kayser

**Affiliations:** 1 Institute of Sports Sciences and Department of Physiology, Faculty of Biology and Medicine, University of Lausanne, Lausanne, Switzerland; 2 Lemanic Neuroscience Doctoral School, University of Lausanne, Lausanne, Switzerland; University of Las Palmas de Gran Canaria, Spain

## Abstract

Hypoxia increases the ventilatory response to exercise, which leads to hyperventilation-induced hypocapnia and subsequent reduction in cerebral blood flow (CBF). We studied the effects of adding CO_2_ to a hypoxic inspired gas on CBF during heavy exercise in an altitude naïve population. We hypothesized that augmented inspired CO_2_ and hypoxia would exert synergistic effects on increasing CBF during exercise, which would improve exercise capacity compared to hypocapnic hypoxia. We also examined the responsiveness of CO_2_ and O_2_ chemoreception on the regulation ventilation (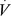
E) during incremental exercise. We measured middle cerebral artery velocity (MCAv; index of CBF), 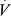
E, end-tidal PCO_2_, respiratory compensation threshold (RC) and ventilatory response to exercise (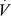
E slope) in ten healthy men during incremental cycling to exhaustion in normoxia and hypoxia (FIO_2_ = 0.10) with and without augmenting the fraction of inspired CO_2_ (FICO_2_). During exercise in normoxia, augmenting FICO_2_ elevated MCAv throughout exercise and lowered both RC onset and
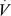
E slope below RC (P<0.05). In hypoxia, MCAv and 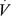
E slope below RC during exercise were elevated, while the onset of RC occurred at lower exercise intensity (P<0.05). Augmenting FICO_2_ in hypoxia increased 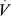
E at RC (P<0.05) but no difference was observed in RC onset, MCAv, or 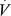
E slope below RC (P>0.05). The 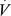
E slope above RC was unchanged with either hypoxia or augmented FICO_2_ (P>0.05). We found augmenting FICO_2_ increased CBF during sub-maximal exercise in normoxia, but not in hypoxia, indicating that the ‘normal’ cerebrovascular response to hypercapnia is blunted during exercise in hypoxia, possibly due to an exhaustion of cerebral vasodilatory reserve. This finding may explain the lack of improvement of exercise capacity in hypoxia with augmented CO_2_. Our data further indicate that, during exercise below RC, chemoreception is responsive, while above RC the ventilatory response to CO_2_ is blunted.

## Introduction

At high intensities of exercise in normoxia, above the respiratory compensation threshold (RC), hyperventilation-induced hypocapnia constricts the cerebral vessels, thereby reducing cerebral blood flow (CBF) and lowering cerebral oxygen delivery [1]–[3]. In conditions of hypoxia, in spite of hypoxic vasodilation, the hypocapnia-induced limitation of CBF at high exercise intensities is further aggravated due to a greater ventilatory drive. Such a limitation of CBF might lead to inadequate cerebral O_2_ delivery, triggering early motor drive withdrawal, thus limiting exercise performance in hypoxia [4], [5]. In support, several studies found a relationship between performance and cerebral deoxygenation in hypoxia using various exercise modes such as repeated sprints [6], incremental exercise [7], [8], and static maximal muscle contraction to exhaustion [9]–[12]. Similarly, under normoxic conditions, exacerbation of exercise induced cerebral deoxygenation, by administering a non-selective beta-blocker, is accompanied by impaired maximal exercise performance [13]. This has led to the hypothesis that an exaggerated hyperventilation-induced hypocapnia and subsequent reduction in CBF during heavy exercise might account, in part, for the impaired performance in hypoxia.

Recently, studies have assessed the effect of preventing the normal drop in end-tidal PCO_2_ on CBF and exercise performance in normoxia [14] and hypoxia [15], [16]. Subudhi et al., [15] reported impaired exercise capacity at 1,600 m and 4,875 m (hypobaric chamber) when they clamped end-tidal PCO_2_ (PETCO_2_) either: 1) at 50 mmHg throughout incremental exercise or 2): at 40 mmHg from ∼75% maximal work rate until exhaustion. Siebenmann et al., [16] completed those observations by investigating, at a more moderate altitude (3,454 m), the impact of clamping PETCO_2_ at 40 mmHg, on performance. Both of these studies found clamping PETCO_2_ increased MCAv and attenuated the decrease in cerebral oxygenation, but slightly decreased peak power output without affecting maximal oxygen uptake. Subudhi et al., [15] found ventilation (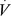
E) was elevated (by ∼50 L/min) during submaximal exercise intensities (37 and 75% of maximal hypoxic work rate) with PETCO_2_ clamping, compared to without added CO_2_. As such, they attributed the reduced performance with CO_2_ clamping to earlier functional limitation by the respiratory system associated with CO_2_ breathing. Since these studies were carried out in subjects living at moderate altitude [15] and in low altitude residents following a day spent at high altitude[16], respectively, both conditions accompanied by enhanced cerebrovascular and ventilatory responsiveness to CO_2_ [17], [18], the acute effect of augmented FICO_2_ and hypoxia on CBF during exercise in an altitude-naïve population remained unclear.

For climbing 8,000 m peaks, a strong ventilatory response at altitude is believed to be a factor for success [19], but increases the risk of brain damage because of excessive hyperventilation-induced cerebral hypoxemia [20]. By contrast, Bernardi et al., [21] found that acclimatized elite climbers with a smaller ventilatory response to hypoxia at 5,200 m were more successful climbing to extreme altitude (Everest and K2), perhaps due to a greater ventilatory reserve (difference between maximal voluntary ventilation and effective minute ventilation) at these altitudes. Therefore, the effect of augmenting FICO_2_ on ventilation during exercise is of interest, particularly in the presence of enhanced ventilatory drive associated with hypoxia.

We tested the hypotheses that, in non-altitude acclimatized subjects, increasing PETCO_2_ during incremental exercise in normobaric hypoxia would: i) further augment CBF during exercise in hypoxia and improve exercise capacity; and ii) exacerbate the increase in ventilation at sub-maximal exercise intensities but not at maximal effort.

## Methods

### Ethics

The study was approved by the Research Ethical Committee of the University Hospitals of Geneva and conformed to the standards set by the Declaration of Helsinki. All subjects were informed regarding the procedures of this study, and informed signed consent was given prior to participation.

### Subjects

Ten healthy male subjects with a mean age of 24±3 yr (mean ± SD), a body mass index of 22.7±2.4 kg/m^2^ and 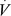
O_2_max of 58.3±10.5 ml/min/kg participated in this study. Subjects were non-smokers, had no previous history of cardiovascular, cerebrovascular, or respiratory disease and were not taking any medication. All the subjects were residents of Geneva, Switzerland (∼400 m) and partook in regular exercise (3–6 h per week); none were acclimatized to altitude (i.e., they had not travelled to altitudes >1,000 m in the past 2 months).

### Experimental design

The subjects visited the laboratory on five occasions. After a full familiarization with the experimental procedures outlined below (visit one), subjects underwent four experimental exercise trials in a randomized, single-blind and balanced fashion (to prevent order effects), with a 3-day washout period between each experimental session, consisting of the following conditions: i) control normoxia (ambient air, 389 m); ii) augmented FICO_2_ in normoxia; iii) control hypoxia (normobaric simulated altitude, 5,000 m); and iv) augmented FICO_2_ in hypoxia. Before each experimental session, the subjects were asked to abstain from caffeine for 12 h and heavy exercise, and from alcohol for 24 h. Each experimental testing session comprised 20 min instrumentation followed by a 4 min resting baseline collection with the subject breathing room-air and seated on an electronically braked cycle ergometer (Ergoselect 100, Ergoline GmbH, Bitz, Germany). During the hypoxia and augmented FICO_2_ trials, the subjects were then switched to a gas mixing system (described below), breathing the respective gas mixtures for an additional 4 min resting baseline (condition baseline) prior to the incremental cycling test.

### Exercise test

The subjects were instructed to begin cycling at 0 watts at a pedaling rate of 70 rpm. The work rate was increased by 0.5 watts every second (30 W/min) thereafter until the subject reached voluntary exhaustion. Throughout each experimental session, the subjects wore a nose-clip and breathed through a mouthpiece attached to a low resistance one-way non-rebreathing valve (Hans-Rudolph 2700, Kansas City, USA). The hypoxia and augmented FICO_2_ were achieved using a modified gas mixing system (Altitrainer, SMTec, Nyon, Switzerland), which has been previously described in detail [14]. In brief, additional CO_2_ was bled into the inspired gas mixture under constant feedback of on-screen PETCO_2_ and FICO_2_ was constantly adapted as to keep PETCO_2_ to a target value of approximately 45 mmHg during both normoxia and hypoxia. The FIO_2_ was held constant at either 0.21 (normoxia, ambient air) or 0.10 (hypoxia). The subjects breathed through the same circuit in all four conditions and were kept unaware to what gas mixture they were breathing and were naive to the rationale of the study. For each subject the experiments were carried out at the same time of day under consistent laboratory conditions (temperature 22.3±0.5°C, humidity 27±7%, barometric pressure 725±4 mmHg).

### Measurements

#### Respiratory variables

Gas exchange was monitored on a breath-by-breath basis (Medgraphics CPX, Loma Linda, USA) measuring flow at the mouth with a Pitot tube and the fractions of inspired and expired O_2_ and CO_2_ with fast responding gas analyzers (infrared and paramagnetic) integrated in the system. Ventilation (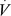
E) was derived from the flow signal and expressed in body temperature and pressure saturated (BTPS) and L/min, respectively. The partial pressure of end-tidal O_2_ (PETO_2_), CO_2_ (PETCO_2_), O_2_ consumption (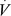
O_2_) and CO_2_ production (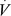
CO_2_) were calculated by the gas analysis system. Prior to each experimental session the system was calibrated using a 3-L syringe (M9474, Medikro Oy, Finland) and precision gas mixtures of known O_2_ and CO_2_ concentrations.

#### Cerebrovascular and cardiovascular variables

Bilateral middle cerebral artery blood flow velocities (MCAv: as index of CBF) were measured in the middle cerebral arteries using a 2-MHz pulsed Doppler ultrasound system (ST3, Spencer technology, Seattle, USA). The Doppler ultrasound probes were positioned over the temporal windows and held firmly in place with an adjustable headband (Marc 600 Headframe, Spencer technology, Seattle, USA). The signals were at depths ranging from 43 to 54 mm. Signal quality was optimized and an M-mode screen shot was recorded to facilitate subsequent probe placements. In our hands, day-to-day reproducibility of MCAv has a coefficient of variation of <10%. The bilateral MCAv were averaged to represent an index of global CBF during rest and exercise. Heart rate was measured using a thoracic belt and recorded directly onto a PC via a wireless receiver pod (PC-POD, Suunto, Vantaa, Finland). Peripheral O_2_ saturation (SpO_2_) was measured from the right ear lobe using pulse oximetry (Satlite, Datex, Helsinki, Finland, and ML320 Oximeter Pod, ADInstruments, Bella Vista, Australia). Blood pressure was measured with an automated arm sphygmomanometer (Ergoselect 100, Ergoline GmbH, Bitz, Germany).

#### Perceived exertion

During exercise, the subjects were asked to score their perceived exercise exertion on the 0–10 Borg scale every minute [22].

### Data and statistical analysis

#### Respiratory compensation threshold and ventilatory response to exercise

The respiratory compensation threshold (RC) was obtained using the v-slope method previously described by Beaver et al., [23]. The assessment of the ventilatory response to exercise (i.e., the slope of 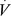
E rise during exercise: 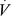
E-slope to power output) has been previously described [24], [25]. In brief, breath-by-breath 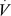
E points were plotted against mechanical power output and a least squares regression was used to determine the 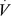
E-slope to exercise above and below RC.

#### Statistical analysis

Resting values were obtained by averaging the data obtained in the last two minutes of the four minutes resting periods just prior to exercise. For the incremental exercise, we took the last 20 sec of every 10% increase in exercise intensity until exhaustion (100%). We then averaged all of the 20 sec segments of the data to obtain a *single mean* value for each variable during all four experimental conditions. The effects of hypoxia and augmenting FICO_2_ on cardiorespiratory and cerebrovascular responses at rest were assessed using two-way repeated-measures ANOVA with α-level of P<0.05 (IBM SPSS Statistics version 21.0, IBM Corporation, Armonk, USA). Likewise, two-way repeated measure ANOVA was used to assess the effects of hypoxia and augmenting FICO_2_ on these variables during incremental exercise. For significant interactions between hypoxia and augmenting FICO_2_, four pairwise comparisons (Bonferroni corrected) were performed to isolate the effect of hypoxia and augmenting FICO_2_ on the dependent measures within subjects with a α-level of 0.0125, indicated where appropriate with the superscript ^B^. Trends were considered when P<0.10. All data are reported as means ± SD.

## Results

All ten subjects completed the experimental protocol. Due to poor gas exchange traces, one subject had to repeat the augmented FICO_2_ in hypoxia trial. Two subjects had to repeat their control normoxia trials, as they initially did not maintain a pedaling rate of 70 rpm during high intensity exercise. One subject reported respiratory dyspnea during exercise in augmented FICO_2_, in hypoxia, while another reported a mild headache lasting 20 min following the control hypoxia trial. No other subjects reported any side effects such as headache or dyspnea following the experiments. Due to poor blood pressure signals during exercise, we were unable to carry out repeated-measures ANOVA analysis on the MAP data during exercise.

### Resting variables (Table 1)

*MCAv*: Hypoxia had no effect on resting MCAv (hypoxia: P = 0.790 vs. normoxia), while augmenting FICO_2_ elevated MCAv by 25±21% during both normoxic and hypoxic conditions (CO_2_: P = 0.001, interaction: P = 0.681).

#### Ventilatory variables

Resting PETCO_2_ was lower with hypoxia (hypoxia: P = 0.001), while it was higher with augmented FICO_2_ (CO_2_: P<0.001, interaction: P = 0.224). There was an interaction between the effects of hypoxia and augmenting FICO_2_ on resting PETO_2_ (interaction: P = 0.004). Post-hoc analysis shows that PETO_2_ was lowered with hypoxia during both control and augmented FICO_2_ conditions (P<0.001^B^ vs. normoxia for both), while augmenting FICO_2_ elevated resting PETCO_2_ by a greater extent in normoxia (by 20±5 mm Hg, P<0.001^B^ vs. control normoxia) compared to in hypoxia (by 10±4 mm Hg, P<0.001^B^ vs. control hypoxia). Resting 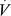
E was elevated by hypoxia (P = 0.008 vs. normoxia) and augmented FICO_2_ (CO_2_: P<0.001, interaction: P = 0.848). There was an interaction between the effect of hypoxia and augmenting FICO_2_ on 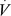
CO_2_ (interaction: P = 0.003). Post-hoc t-tests showed that hypoxia elevated 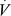
CO_2_ during control (P<0.001^B^ vs. control normoxia) but not during augmented FICO_2_ (P = 0.832^B^ vs. augmented FICO_2_ in normoxia). Accordingly, 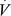
CO_2_ appeared to be lower with augmented FICO_2_ in hypoxia (P<0.001^B^ vs. control hypoxia) but not in normoxia (P = 0.131^B^ vs. control normoxia). No differences were observed in 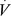
O_2_ with either hypoxia or augmented FICO_2_ (main effects: P = 0.217 & P = 0.127, respectively, interaction: P = 0.575).

#### Cardiovascular variables

Hypoxia elevated resting HR (hypoxia: P<0.001 vs. normoxia); there was a slight but non-significant tendency for augmenting FICO_2_ to attenuate this increase in HR (CO_2_: P = 0.886, interaction: P = 0.059). The effect of hypoxia and augmented FICO_2_ on resting SpO_2_ interacted with each other (interaction: P = 0.004). Specifically, post-hoc analysis found SpO_2_ to be lower with hypoxia during both control and augmented FICO_2_ conditions (P<0.001^B^ vs. normoxia for both). Meanwhile, augmenting FICO_2_ selectively elevated resting SpO_2_ in hypoxia (P<0.001^B^ vs. control hypoxia), but not in normoxia (P = 0.615^B^ vs. control normoxia). No changes were observed in resting MAP with either hypoxia or augmenting FICO_2_ (main effects: P = 0.809 and P = 0.123, respectively, interaction: P = 0.370).

### Exercise

#### Performance

Hypoxia lowered maximal exercise capacity by 26% (251±15 W vs. 338±21 W, hypoxia vs. normoxia, hypoxia: P = 0.001), whereas there was a tendency for maximal exercise capacity to be lowered with augmented FICO_2_ (CO_2_: P = 0.091, interaction: P = 0.397). Specifically, maximal exercise capacity tended to be lower by 4% in normoxia (332±66 W vs. 345±66 W, augmented FICO_2_ vs. control, P = 0.052^B^), while no trend was observed in hypoxia (248±48 W vs. 256±48 W, P = 0.252^B^).

#### Ventilatory variables (Fig. 1A, 1B & 2A–D)

During incremental exercise, the effect of augmenting FICO_2_ on PETCO_2_ was greater in hypoxia compared to normoxia (interaction: P<0.001). Post-hoc analysis revealed that throughout exercise, hypoxia lowered PETCO_2_ during control by a greater extent than during augmented FICO_2_ (10±3 mmHg vs. 5±3 mmHg, P<0.001^B^ vs. normoxia for both). Meanwhile, augmenting FICO_2_ elevated PETCO_2_ by a lesser extent in normoxia compared to in hypoxia (8±3 mmHg vs. 14±3 mmHg, P<0.001^B^ vs. control for both). In contrast, hypoxia blunted the effect of augmenting FICO_2_ on PETO_2_ (interaction: P<0.001). Post-hoc comparisons found hypoxia lowered PETO_2_ by 49±2 mmHg during control and by 54±4 mmHg during augmented FICO_2_ conditions (P<0.001^B^ vs. normoxia for both). Furthermore, augmenting FICO_2_ increased PETO_2_ by 15±3 mmHg in normoxia and by 10±3 mmHg in hypoxia (P<0.001^B^ vs. control for both).

During incremental exercise, both hypoxia and augmenting FICO_2_ elevated 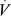
E (main effect: P<0.001 for both, interaction: P = 0.188). At exhaustion, 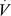
E reached 74±12% of estimated maximal voluntary ventilation (MVV) in normoxia and 69±11% in hypoxia [26].

#### MCAv (Fig. 1C, 2E & 2F)

During incremental exercise, there was a significant interaction between the effect of hypoxia and augmenting FICO_2_ (interaction: P = 0.010). Post-hoc analysis revealed that hypoxia elevated MCAv during control (P = 0.002^B^ vs. control normoxia), but not during augmented FICO_2_ condition (P = 0.429^B^ vs. augmented FICO_2_ in normoxia). Meanwhile, augmenting FICO_2_ selectively elevated MCAv during incremental exercise in normoxia (P<0.001^B^ vs. control normoxia), but not in hypoxia (P = 0.145^B^ vs. control hypoxia).

#### Oxygen saturation (Fig. 1D, 2G & 2H)

The effect of augmenting FICO_2_ on SpO_2_ during exercise was enhanced in hypoxia (interaction: P<0.001). As such, post-hoc analysis showed that hypoxia lowered SpO_2_ by 26±6% during control condition (P<0.001^B^ vs. control normoxia) and by 15±6% during augmented FICO_2_ condition (P<0.001^B^ vs. augmented FICO_2_ in normoxia). Meanwhile, augmenting FICO_2_ elevated SpO_2_ by 12±5% in hypoxia (P<0.001^B^ vs. control hypoxia), but not in normoxia (P = 0.615^B^ vs. control normoxia).

#### Metabolism (Fig. 1E, 1F & 3A–D)

Hypoxia lowered both 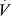
O_2_ and 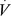
CO_2_ throughout exercise (hypoxia: P<0.001 vs. normoxia for both), while augmented FICO_2_ had no effect on these variables (CO_2_: P = 0.628 & P = 0.544, respectively, interaction: P = 0.954 & P = 0.304).

#### Cardiovascular variables (Fig. 1G, 3E & 3F)

During exercise, the effect of hypoxia on HR was offset by the effect of augmenting FICO_2_ (interaction: P = 0.003). Accordingly, post-hoc t-tests found HR to be higher with hypoxia during control (P = 0.001^B^ vs. control normoxia), but not during the augmented FICO_2_ (P = 0.360^B^ vs. augmented FICO_2_ in normoxia). There were trends for augmenting FICO_2_ to elevate HR during exercise in normoxia (P = 0.075^B^ vs. control normoxia) and lower HR in hypoxia (P = 0.019^B^ vs. control hypoxia).

#### Rate of perceived exertion (Fig. 1H, 3G & 3H)

There was an interaction between effects of hypoxia and augmenting FICO_2_ on RPE (interaction: P = 0.045). As a result, a post-hoc t-tests found a tendency for RPE to be elevated with augmented FICO_2_ in normoxia (P = 0.038^B^ vs. control normoxia), but not in hypoxia (P = 0.598^B^ vs. control hypoxia). Hypoxia had no effect on RPE during control or augmenting FICO_2_ (P = 0.735^B^ & P = 0.180^B^ vs. normoxia, respectively).

#### RC and 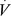
E-slope to exercise (Fig. 4 & 5)

During exercise, the onset of RC occurred at lower power output in hypoxia compared to in normoxia (by 86±36 W, Hypoxia: P<0.001). Likewise, the onset of RC occurred at lower power output with augmenting FICO_2_ compared to control conditions (CO_2_: P = 0.015; interaction: P = 0.176). Meanwhile, there was an interaction between the effect of hypoxia and augmenting FICO_2_ on 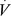
E at RC (interaction: P = 0.021). Post-hoc analysis revealed that hypoxia tended to elevate 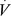
E at RC under the augmented FICO_2_ condition (P = 0.027^B^ vs. augmented FICO_2_ in normoxia), but not during the control condition (P = 0.112^B^ vs. control normoxia). Augmenting FICO_2_ selectively increased 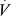
E at RC in hypoxia (by 23.95±21.70 L/min, P = 0.007^B^ vs. control hypoxia), but not in normoxia (P = 0.847^B^ vs. control normoxia). The effect of hypoxia and augmenting FICO_2_ interacted with each other on the 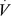
E-slope to exercise (the slope of 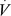
E rise against power) below RC (interaction: P = 0.043). As such, hypoxia elevated the 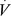
E slope to exercise below RC during both control (by 30±13%, P<0.001^B^ vs. control normoxia) and augmented FICO_2_ condition (by 93±78%, P = 0.001^B^ vs. augmented FICO_2_ in normoxia). Augmenting FICO_2_ lowered the 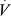
E slope below RC during normoxia (by 27±14%, P = 0.001^B^ vs. control normoxia), but not during hypoxia (P = 0.786^B^ vs. control hypoxia). In contrast, no differences were observed in the 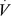
E slope to exercise above RC with either hypoxia or augmenting FICO_2_ (hypoxia: P = 0.256, CO_2_: P = 0.155, interaction: P = 0.585).

## Discussion

**Figure 1:**
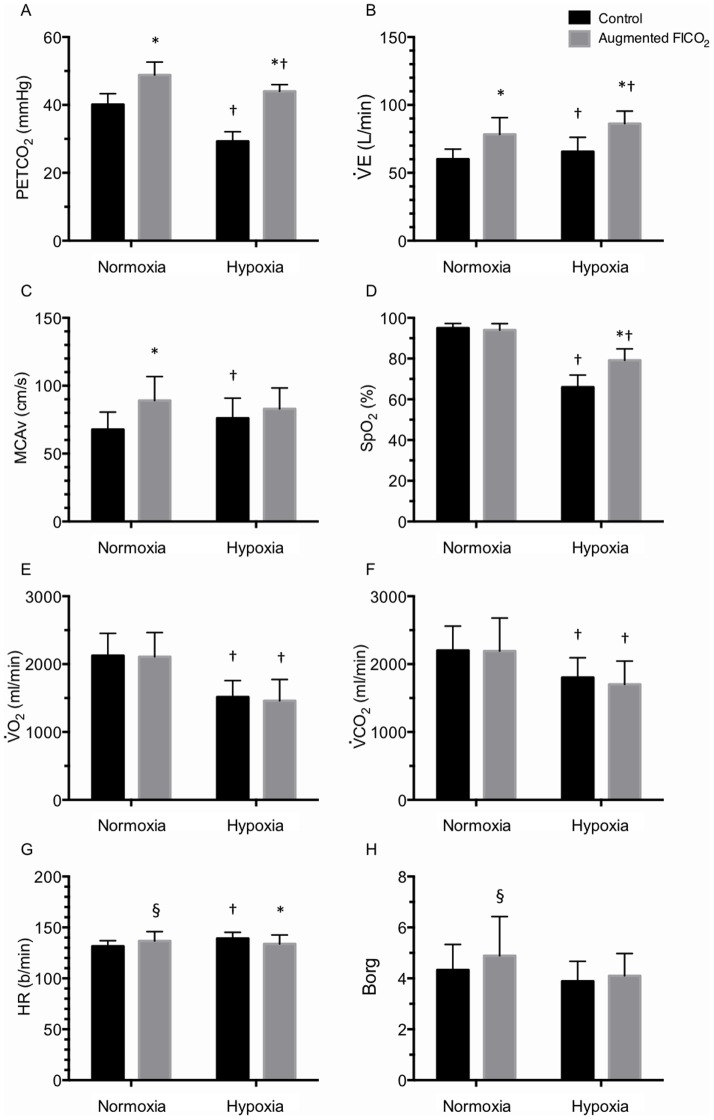
Effect of hypoxia and augmented FICO_2_ on mean respiratory, cerebrovascular, metabolic and cardiac variables and perceived effort of exertion during incremental cycling to exhaustion. Values expressed as mean ± SD. * different from control (P<0.05); † different from normoxia (P<0.05); § trend for a difference (P<0.10).

**Figure 2:**
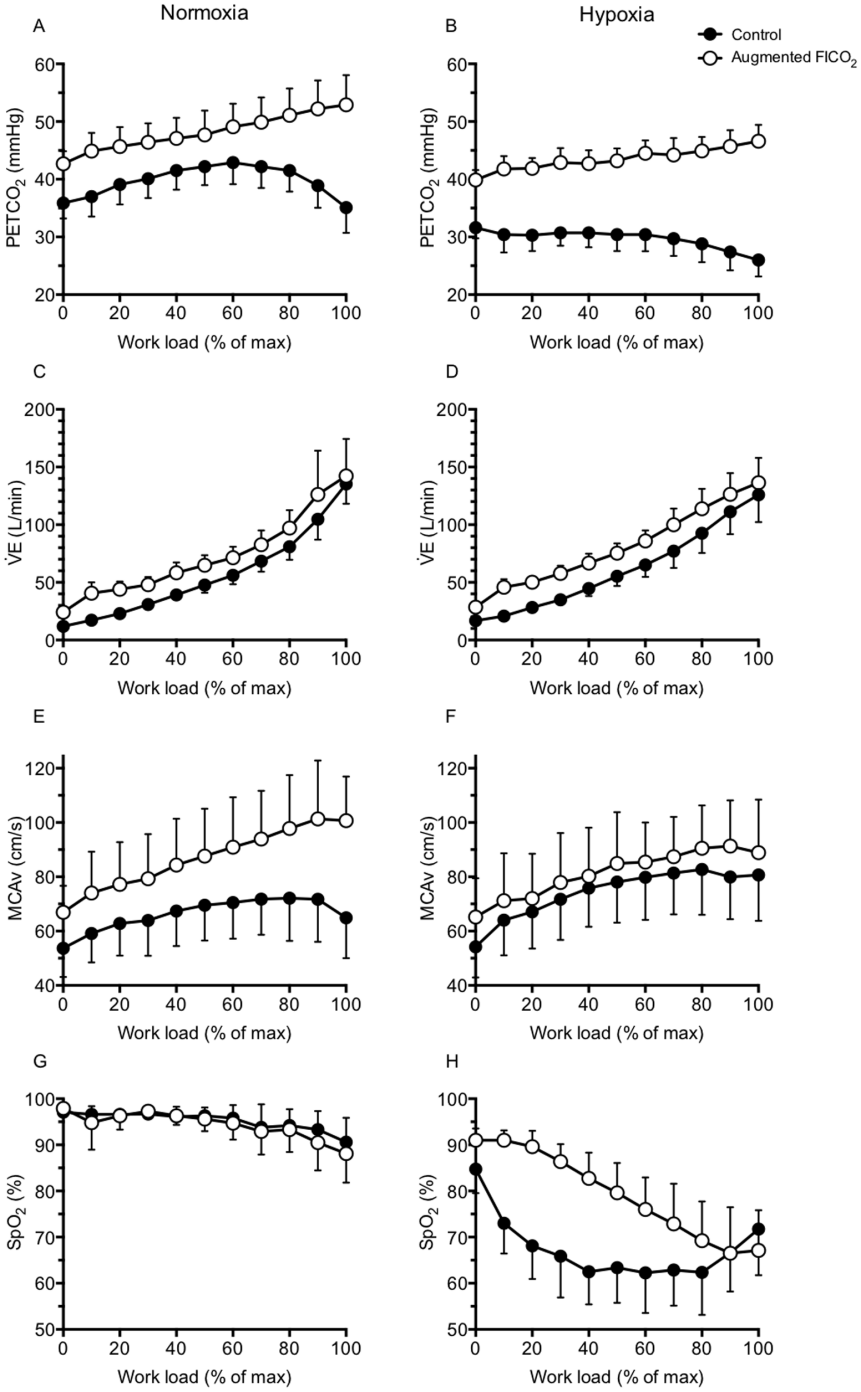
Effect of hypoxia and augmented FICO_2_ on group respiratory, cerebrovascular variables and peripheral O_2_ saturation during incremental cycling to exhaustion. Left panels: group data in normoxia (mean ± SD); right panels: group data in hypoxia. Note: these graphs are only intended for visualizing the changes in physiological parameters during incremental cycling. Statistical analyses were carried out using the average variable during the exercise session (see Figure 1).

Augmenting FICO_2_ elevated CBF during incremental exercise in normoxia, but, unexpectedly, it did not during hypoxia. Furthermore, we found no improvement in exercise capacity with augmented FICO_2_ during either normoxia or hypoxia. While the roles of hypercapnia and hypoxia in the regulation of CBF during rest have been extensively studied, their effects on cerebrovascular control during exercise are less well documented [see [27] for review]. During exercise, studies have found enhanced CBF response to hypercapnia [28], while the CBF response to hypocapnia remained unchanged [29]. During incremental exercise, we observed higher MCAv during both control hypoxia and augmented FICO_2_ in normoxia (Fig. 1C). However, in contrast to the findings by Subudhi et al., [15], we observed no further increase in MCAv with augmented FICO_2_ in hypoxia. The discrepancies between these findings could potentially be accounted for by the differences in the experimental setup and/or the level of hypercapnia achieved. In the study by Subudhi et al., [15], PETCO_2_ was clamped at 50 mmHg throughout exercise, while our subjects' PETCO_2_ were on average, ∼46 and ∼43 mmHg in normoxia and hypoxia respectively (Fig. 1A), reaching ∼53 and ∼47 mmHg at maximal exercise intensity (Fig. 2A & 2B). Meanwhile, using our Altitrainer setup, Siebenmann et al., [16] were able to increase MCAv during incremental exercise in moderate hypobaric hypoxia (3,454 m) by clamping PETCO_2_ at 40 mmHg in altitude sojourners. We deem it unlikely that differences in experimental setup are sufficient to account for these discrepant findings between the three studies. An alternative explanation is the potential influence of (partial) altitude acclimatization [30]. Subudhi et al., [15] examined the effect of CO_2_ clamping on competitive cyclists living and training at 1,650 m, therefore already acclimatized to exercise at moderate altitude. Likewise, the subjects in Siebenmann et al., [16] had spent one night at altitude. In contrast, the subjects in our study had not been exposed to altitude >1000 m in the 2 months prior the study. Since acclimatization to altitude augments the cerebrovascular responsiveness to CO_2_ [17], the increase in CBF observed by both Subudhi et al., [15] and Siebenmann et al., [16] may therefore be due to a partial state of acclimatization to altitude.

### Cerebral O_2_ delivery and performance in hypoxia

**Figure 3:**
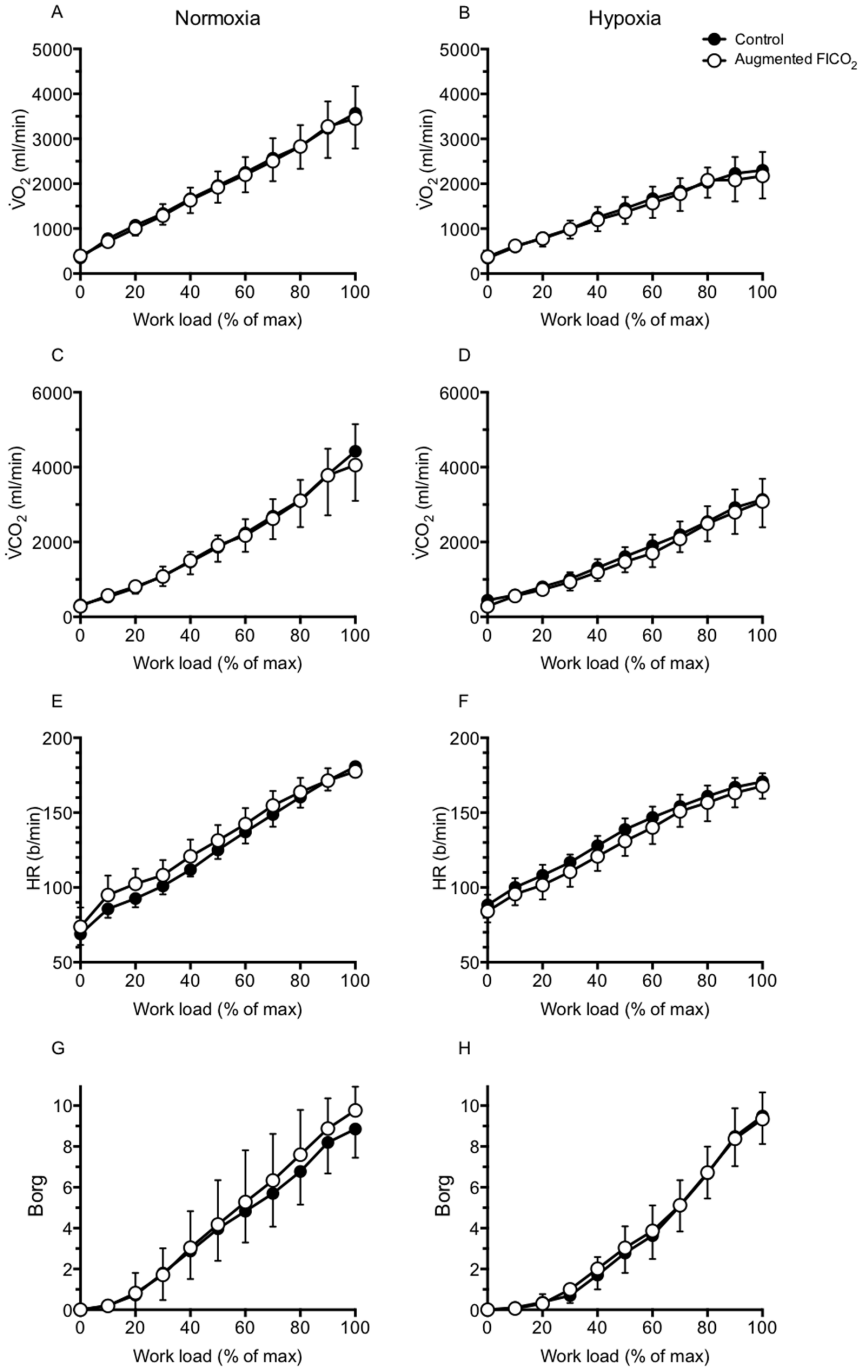
Effect of hypoxia and augmented FICO_2_ on metabolic variables, heart rate, and perceived effort of exertion during incremental cycling to exhaustion. Left panels: group data in normoxia (mean ± SD); right panels: group data in hypoxia. Note: these graphs are only intended for visualizing the changes in physiological parameters during incremental cycling. Statistical analyses were carried out using the average variable during the exercise session (see Figure 1).

Contrary to our first hypothesis, augmenting FICO_2_ during incremental exercise in normobaric hypoxia failed to improve aerobic exercise capacity. This finding corroborates recent findings in milder (hypobaric) hypoxic conditions [15], [16]. The relationship between cerebral deoxygenation and exercise performance in normoxia [13] and hypoxia [6]–[12] is therefore unlikely to be causally related to the loss of aerobic capacity in hypoxia. However, it cannot be excluded that augmenting FICO_2_ introduces limits that overrule the potential benefit of improved cerebral CO_2_ delivery. Siebenmann et al., [16] argued that inspired CO_2_ may exacerbate the metabolic acidosis associated with heavy exercise, shifting the oxyhemoglobin curve rightward, decreasing SaO_2_, thus limiting peak 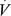
O_2_. In agreement, our results show a reduction in average heart rate in hypoxia (Fig. 1G) and a tendency for maximal exercise capacity to be actually impaired with augmented FICO_2_ (P = 0.091), as also reported elsewhere [15], [16]. In agreement with Subudhi et al., [15], we found the participants' RPE to increase more rapidly with augmented FICO_2_ in normoxia (Fig. 3G), in parallel with the increase in ventilation (Fig. 2C). But contrary to Siebenmann et al., [16], our findings of higher SpO_2_ with augmented FICO_2_ in hypoxia throughout submaximal exercise intensities, and similar SpO_2_ at exhaustion (Fig. 2H), which exclude a right-ward shift in the oxyhemoglobin curve would have limited pulmonary oxygen uptake.

### Limits to cerebral vasodilation

While controversy remains, there is a large body of literature suggesting that nitric oxide, prostanoids and C-natriuretic peptide are involved in the cerebral vasodilatory responses to both hypercapnia and hypoxia through a number of intermediate interacting/co-activating pathways [31]. We speculate that if one chemical stimulus, such as hypoxia, is of sufficient magnitude to exhaust the cerebral vessels' capacity to vasodilate, thereby reaching maximal diameter of the vessels, then any additional stimuli would have no further effect on cerebrovascular resistance. Our data indicate that the CBF response to hypoxia is enhanced during exercise compared to rest, while adding CO_2_ to the inspired gas during exercise only elevates CBF during normoxia, but not during severe hypoxia (Fig. 1C). The ‘normal’ cerebrovascular reactivity to hypercapnia thus appears to be abolished during exercise in severe hypoxia (Fig. 2F). In support, we found no difference between the MCAv values between augmented FICO_2_ in normoxia and control hypoxia (P = 0.193). Such exhaustion of the cerebral vessels' vasodilatory reserve would account for the lack of change in MCAv with augmented FICO_2_ during sub-maximal exercise in hypoxia. Mardimae et al., [32] demonstrated a synergistic role of CO_2_ and hypoxia on the control of CBF at rest whereas our results would favor a negative effect, whereby the presence of severe hypoxia appears to attenuate the effect of hypercapnia on the cerebral vessels. Our data indicates that during incremental exercise in hypoxia, the role of hypercapnia in the regulation of cerebrovascular tone appears to be diminished – at least in an unacclimatized population. However, we cannot exclude the possibility that there might be regional differences in the cerebral vessel response to hypoxia and hypercapnia, which could display additive or synergistic interactions between the two chemical stimuli. Furthermore, the effect of combining hypoxia and hypercapnia on cerebral metabolism is unknown. Given the limited literature on this topic, further studies on the effect of hypoxia, hypercapnia and acclimatization status on regulation of cerebrovascular tone and therefore cerebral O_2_ delivery during exercise in altitude is certainly warranted.

### Chemoreception and exercise hyperpnea

**Figure 4:**
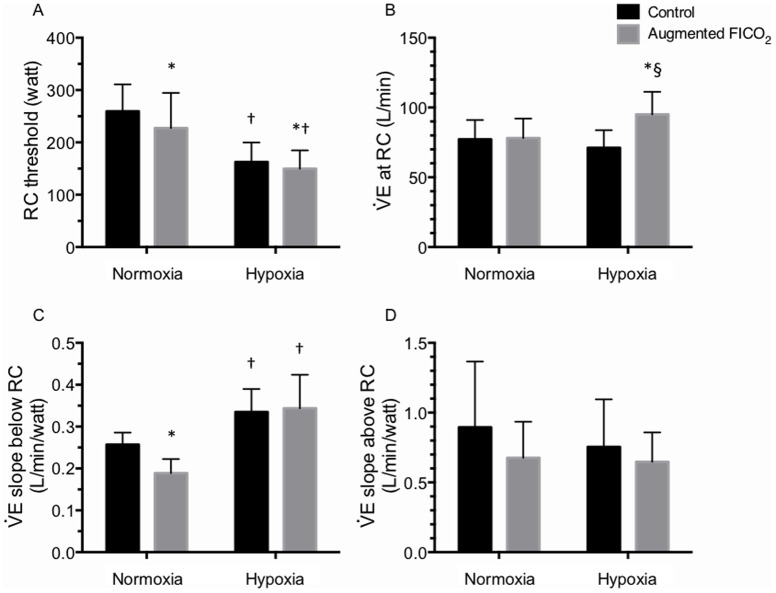
Effect of hypoxia and augmented FICO_2_ on respiratory compensation threshold and ventilatory response to exercise during incremental cycling to exhaustion. Values expressed as mean ± SD. * different from control (P<0.05); † different from normoxia (P<0.05); § trend for a difference (P<0.10).

**Figure 5:**
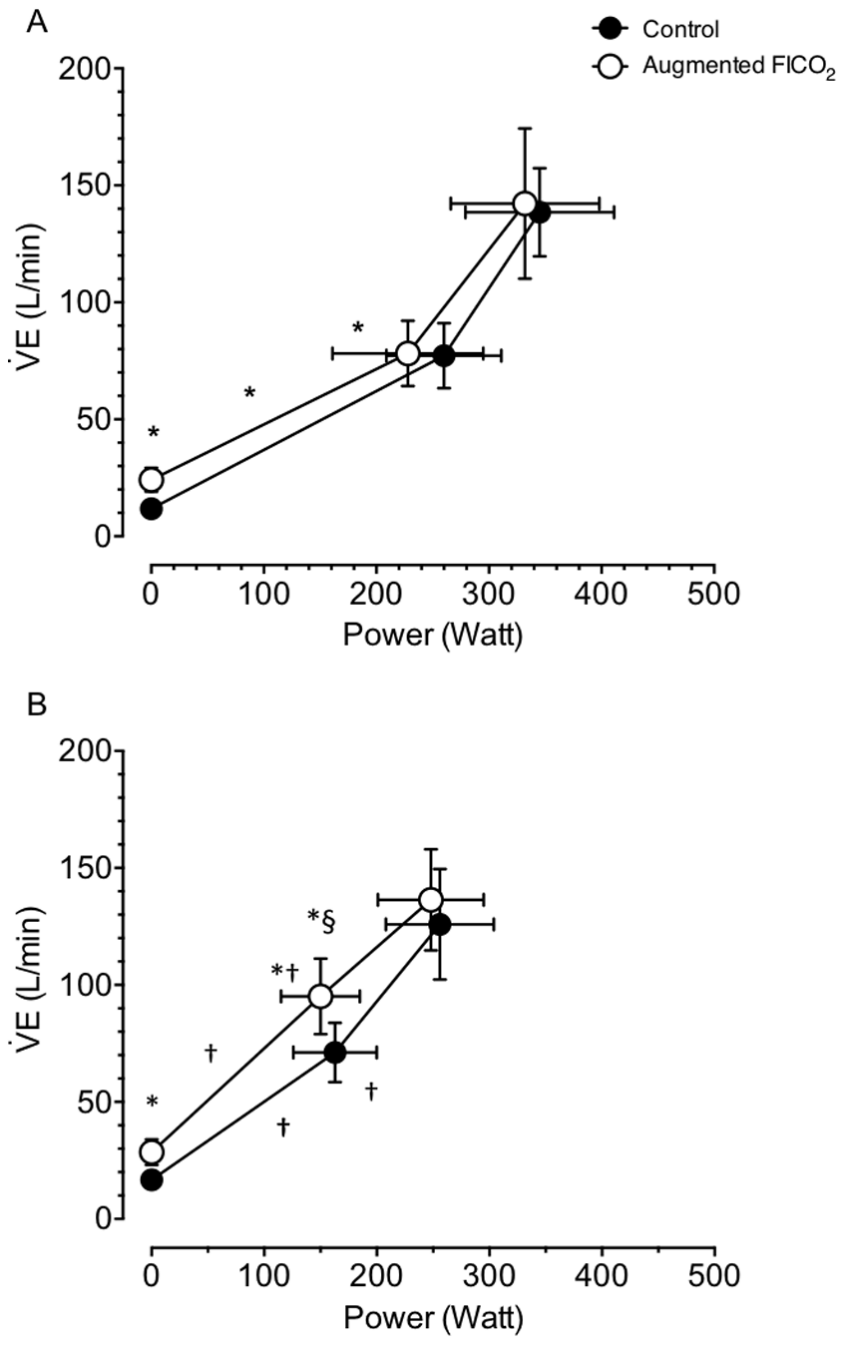
Effect of hypoxia and augmented FICO_2_ on respiratory compensation threshold and ventilatory response to exercise during incremental cycling to exhaustion. A: group data in normoxia (mean ± SD); B: group data in hypoxia. * different from control (P<0.05); † different from normoxia (P<0.05); § trend for a difference (P<0.10).

The regulation of exercise hyperpnea has been extensively studied during the past 100 years [see [33]–[37] for reviews]. Nevertheless, the role of chemoreception in the regulation of exercise hyperpnea, especially during heavy exercise remains controversial [38], [39]. To date, only a handful of studies have examined the effect of chemoreceptor stimulation with CO_2_ alone [3], [14], [25], [40], [41] or the combined effects of hypoxia and hypercapnia on exercise hyperpnea [42]. Our second hypothesis stated that increasing PETCO_2_ and decreasing PETO_2_ would stimulate the chemoreceptors and lead to an increase in the ventilatory response to exercise. We found that hypoxia and augmented FICO_2_ had an additive effect on exercise hyperpnea below the RC threshold (Fig. 4C). As reported before in conditions of normoxia [14], adding CO_2_ to the inspirate during normoxic and hypoxic conditions indeed increased ventilation at rest and during incremental exercise (Table 1 & Fig. 5). However, at higher intensities, above the RC threshold, this effect progressively lessened, in spite of a progressive increase in PETCO_2_, again similarly to what we found before in normoxia [14]. Hypoxia *per se* augmented the ventilatory response to incremental exercise (the rate of ventilation increase during exercise) below the RC threshold, while augmented FICO_2_ attenuated this rate of increase (Fig. 5). Hypoxia shifted the RC threshold to lower exercise intensities, which is further exacerbated with augmented FICO_2_ (Fig. 4A & 5B). No differences were observed in the slope of the ventilatory response to exercise above the RC threshold between any of the conditions (Fig. 4D). We thus confirm that below the RC threshold both hypoxia and hypercapnia can modulate the rate of ventilatory response to increasing exercise, presumably through chemoreceptor stimulation. However, once above the ventilatory compensation threshold, the chemoreflex ventilatory responses are blunted.

**Table 1:** Effect of hypoxia and augmented FICO_2_ on resting respiratory, cerebrovascular and cardiovascular variables.

	Normoxia						Hypoxia					
	Control			Augmented FICO_2_			Control			Augmented FICO_2_		
**_Ventilatory_**												
PETCO_2_ (mmHg)	36	±	3	43	±	2*	32	±	2†	40	±	2* †
PETO_2_ (mmHg)	96	±	4	116	±	4*	45	±	3†	55	±	3* †
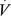 E (L/min)	11.6	±	1.8	24.1	±	5.1*	16.5	±	2.4†	28.5	±	5.5* †
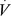 O_2_ (ml/min)	350	±	64	394	±	63	346	±	91	375	±	75
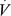 CO_2_ (ml/min)	320	±	67	285	±	51	444	±	85†	282	±	66*
**Cerebrovascular**												
MCAv (cm/s)	54	±	10	67	±	10*	54	±	12	66	±	16*
**Cardiovascular**												
HR (b/min)	70	±	7	74	±	13	88	±	7	84	±	7*
MAP (mmHg)	90	±	7	99	±	11	90	±	14	100	±	9
SpO_2_ (%)	97	±	1	98	±	1	85	±	5†	91	±	3* †

Values are mean ± SD.

*different from control (P<0.05);

† different from normoxia (P<0.05).

N = 10 for all variables except VO_2_ and VCO_2_ where N = 9.

Siebenmann et al., [16] suggested that, during heavy exercise in hypoxia, the hyperventilation-induced hypocapnia might attenuate ventilatory drive and limit the ventilatory response to exercise. They further speculated that the mechanical constraints associated with high intensity exercise ventilation may be reduced in hypobaric hypoxia, while at the same time hypocapnia would be more pronounced, so that a blunting effect of hypocapnia on 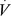
E would persist or become more pronounced. Since we performed our experiments in normobaric conditions our results were not influenced by differences in air density, neglecting the small increases in N_2_ and CO_2_ in exchange of O_2_ in our inspirates. In our young and active, but not-athletic subjects, mechanical limitation would seem unlikely since maximal ventilation only reached ∼70% of estimated MVV with augmented FICO_2_ both in normoxia and hypoxia.

### Methodological considerations

Although the present study provided the opportunity to examine the effects of augmented FICO_2_ on CBF response and exercise capacity during incremental exercise in hypoxia, an important limitation of the present study, as of many other studies, is the assumption that MCAv represents global CBF changes. Sato et al., [43] recently found that blood flow in the middle cerebral and internal carotid arteries declined from moderate (60% 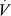
O_2_max) to high intensity exercise (80% 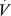
O_2_max), whilst blood flow in the vertebral artery continued to increase at the higher intensities. This led Vogiatzis et al., [11] to suggest that vertebral artery blood flow may compensate for reductions of MCA blood flow during near-maximal or maximal exercise. However, since the relative contribution of the internal carotid artery (and therefore MCA) to global CBF remains relatively unchanged (∼65%) at high intensity exercise [43], we contend that MCAv is a reasonable index of changes in global CBF during heavy exercise. Further consideration when interpreting our MCAv data is the potential influence of hypoxia and hypercapnia on the MCA diameter. The cross-sectional area of the MCA was shown to remain relatively unchanged within a wide range of changes in PETCO_2_ [44]–[47] and during exposure to hypoxia similar to that used in the present study (5,300 m) [48]. However, since no studies have examined the effect of exercise on MCA diameter, nor when superimposed with hypoxia and hypercapnia, we cannot exclude the possibility MCA diameter might have increased in our study, leading to an underestimation of the effect of augmented FICO_2_ or hypoxia on CBF. In addition, since we did not measure cerebral tissue oxygenation, it is possible that we were unable to elevated cerebral O_2_ delivery with our experimental setup. However, in the present study, SpO_2_ was elevated with augmented FICO_2_ throughout exercise in hypoxia (Fig. 1D), while MCAv was comparable between the control and augmented FICO_2_ conditions (Fig. 1C). We would therefore expect cerebral O_2_ delivery, which is the product of arterial O_2_ saturation and CBF, to be elevated with augmented FICO_2_ in hypoxia, leading to increased cerebral tissue oxygenation.

Another limitation of the present study is that we increased end-tidal rather than arterial PCO_2_ during exercise, while it is known that the end-tidal-arterial PCO_2_ gradient [49] varies with exercise [50], [51]. Using our setup, Siebenmann et al., [16] were successful in clamping both end-tidal and arterial PCO_2_ at around 40 mmHg during incremental exercise in hypoxia. Therefore, we believe it is likely that we were able to sufficiently elevate PaCO_2_.

Finally, in the present study, we used a linear ramp protocol to assess the effect of augmented FICO_2_ on the ventilatory response during incremental exercise. It should be acknowledged that during incremental exercise, lower peak 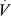
O_2_ and work output is obtained during ramp vs. step protocols [52], while the steepness of the ramp (i.e., 10 W/min, 30 W/min or 50 W/min), pedaling frequency and baseline workload can influence the onset of ventilatory threshold, peak 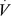
O_2_ and the 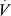
O_2_-workload relationship [53], [54]. Therefore, our conclusions on the ventilatory response to exercise should only be limited to the specific ramp protocol (30 W/min) used in the present study. Furthermore, since all the aforementioned studies [15], [16] only looked at aerobic capacity using incremental exercise tests until voluntary exhaustion, which have limited ecological validity [55], it remains to be investigated if increasing MCAv by augmenting FICO_2_ during steady-state sub-maximal endurance exercise such as a time trial could improve performance in hypoxia, as time trial exercise provide a more accurate simulation of physiological responses during actual competitions [56] and correlate well with actual race performance [57].

## Conclusions

We report two novel findings. Firstly, augmenting FICO_2_ increases cerebral blood flow during sub-maximal exercise in normoxia, but not in hypoxia. This finding indicates that the ‘normal’ cerebrovascular response to hypercapnia is impaired during exercise in hypoxia, possibly due to an exhaustion of cerebral vasodilatory reserve. Secondly, above the ventilatory recruitment threshold, both in normoxia and hypoxia, the chemoreflex ventilatory responses are blunted.
